# Endoscopic treatment of sagittal suture synostosis — a critical analysis of current management strategies

**DOI:** 10.1007/s10143-022-01762-y

**Published:** 2022-04-06

**Authors:** Verena Fassl, Laura Ellermann, Gabriele Reichelt, Phillipe Pape, Christoph Blecher, Christian Hoffmann, Florian Ringel, Bilal Al-Nawas, Julia Heider, Malte Ottenhausen

**Affiliations:** 1 Department of Neurological Surgery, University Medical Center Mainz, Langenbeckstr. 1, 55131 Mainz, Germany; 2 Department of Anesthesiology, University Medical Center Mainz, Mainz, Germany; 3 Department of Pediatrics, University Medical Center Mainz, Mainz, Germany; 4 Cranioform, Berlin, Germany; 5 Department of Pediatric Radiology, University Medical Center Mainz, Mainz, Germany; 6 Department of Oral and Maxillofacial Surgery, University Medical Center Mainz, Mainz, Germany

**Keywords:** Craniosynostosis, Endoscopic, Endoscopically assisted, Suturectomy, Sagittal suture, Scaphocephalus

## Abstract

While many centers nowadays offer minimally invasive techniques for the treatment of single suture synostosis, surgical techniques and patient management vary significantly. We provide an overview of how scaphocephaly treated with endoscopic techniques is managed in the reported series and analyze the crucial steps that need to be dealt with during the management process. We performed a review of the published literature including all articles that examined sagittal-suture synostosis treated with endoscopic techniques as part of single- or multicenter studies. Fourteen studies reporting results of 885 patients were included. We identified 5 key steps in the management of patients. A total of 188 patients were female and 537 male (sex was only specified in 10 articles, for 725 included patients, respectively). Median age at surgery was between 2.6 and 3.9 months with a total range from 1.5 to 7.0 months. Preoperative diagnostics included clinical and ophthalmologic examinations as well as neuropsychological and genetic consultations if needed. In 5 publications, a CT scan was routinely performed. Several groups used anthropometric measurements, mostly the cephalic index. All groups analyzed equally recommended to perform endoscopically assisted craniosynostosis surgery with postoperative helmet therapy in children < 3 months of age, at least for non-syndromic cases. There exist significant variations in surgical techniques and patient management for children treated endoscopically for single suture sagittal synostosis. This heterogeneity constitutes a major problem in terms of comparability between different strategies.

## Introduction

The premature fusion of single cranial sutures results in craniofacial deformities. While cranial vault remodeling through large skin incisions has been used and evolved for decades [19, 36], the endoscopic techniques were introduced more recently in the early 1990s by Jimenez and Barone [27]. Endoscopy-assisted suturectomies have proven to result in cosmetic outcomes similar to those achieved by open approaches [2, 5, 12, 17, 18, 20–22, 29], whereas the published data suggests that operating room times, length of hospital stay, and rates of blood transfusions were reduced [10, 11, 13, 15, 49]. Sagittal suture synostosis, depicting the most common form of craniosynostosis, accounts for 40–60% of all single suture synostosis [26]. Among these, anterior sagittal suture closure with frontal bossing, posterior sagittal suture closure with an occipital knob or bathrocephaly, as well as complete sagittal synostosis have to be differentiated [26].

While most centers nowadays offer minimally invasive techniques for treating single suture synostosis, surgical techniques and patient management vary significantly between the different centers [16]. Over the past years, this heterogeneity in data presentation made it almost impossible to compare the different approaches used by multiple groups in a sophisticated, evidence-based manner. This led to our research question on which consistent points in the current published data referring to the endoscopic management of patients with SCS could be based on elaborated process to be used more commonly facilitating an evidence-based comparison between the craniofacial centers in the future. Therefore, by conducting this review, we want to provide an overview of how scaphocephaly treated with endoscopic techniques is managed in the reported series and also analyze the crucial steps that need to be dealt with during the management process.

## Methods

### Literature search strategy

We performed a systematic review of the published literature to identify relevant articles. The data collection was retrieved by online searches through PubMed and MEDLINE, respectively, in compliance with the Preferred Reporting Items for Systematic Reviews (PRISMA) conducted in July 2021. Search terms used were “sagittal suture craniosynostosis,” “endoscopic suturectomy,” and “endoscopy-assisted suturectomy” in the period between 1961 and 2021. In addition to that, we found articles by further exploring the reference lists of publications initially identified.

The articles were initially selected based on a first review of the titles and abstracts using predetermined inclusion and exclusion criteria. We defined our inclusion criteria as English language articles presenting case series that referred to single-suture sagittal craniosynostosis treated with endoscopic techniques as part of single- or multiple-center studies providing adequate topicality. Exclusion criteria consisted of complex or multiple-suture craniosynostosis, open surgery for treatment, editorials, and previously existing systematic reviews and meta-analysis since we aimed to provide an objective overlook and analysis of the current management. After an independent full-text review by the authors, final inclusion was determined by consensus.

In total, we initially identified 695 publications, wherein 135 articles meeting the inclusion criteria were retrieved for further screening. After having evaluated the full texts, 121 articles were excluded because of inconsistency in study design, reproducible overlap between samples, or heterogenic data, including complex and/or multiple suture craniosynostosis not obviously mentioned at first sight. After close evaluation, 14 articles published between 2011 and 2021 remained and were finally used for quantitative and qualitative data extraction. Although we were aware of a sample overlap between two of the major groups [37, 44], we consciously decided to analyze the data nevertheless, as we did not want to forego the essential insights gained by the analysis of such a vast number of patients that otherwise would fit perfectly in our inclusion criteria. Since we were not able to reliably figure out how many, respectively, which cases have been investigated in both publications, this sure might be a point of criticism regarding our work. However, we had to counterbalance that fact with the benefit of learning from additional data.

**Fig. 1:**
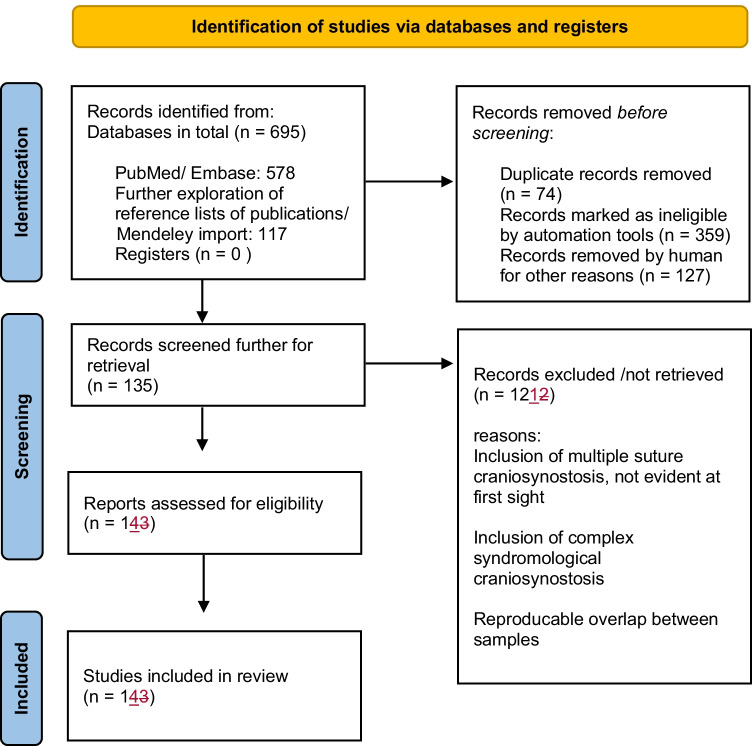
A flow diagram illustrating our literature research based on PRISMA criteria. Modified from: Page MJ, McKenzie JE, Bossuyt PM, Boutron I, Hoffmann TC, Mulrow CD, et al. The PRISMA 2020 statement: an updated guideline for reporting systematic reviews. BMJ 2021;372:n71. doi: 10.1136/bmj.n71

Figure 1 shows a flow chart illustrating our literature research based on PRISMA criteria.

### Data extraction

The data extracted comprised, whenever possible: consistency of the multidisciplinary specialized team, preoperative examinations and measurements, anthropometric and demographic data of the patient samples, specification of the surgical procedure including its corresponding size and location of incisions and craniotomies, intra- and perioperative management and complications, perioperative parameter (e.g., duration of surgery, estimated blood loss), postoperative outcome measures, postoperative therapy, cost analysis, and follow-ups. All data was analyzed with descriptive statistics.

## Results

Most studies included were retrospective in design, whereby two authors [28, 44] reported on their prospectively analyzed sample. The number of patients presented varied between 5 and 256 [6, 28, 35]. In total, 885 children with isolated sagittal suture craniosynostosis were included, wherein 188 were female and 537 male (sex was only specified in 10 articles, 725 included patients respectively). Their median age at surgery was between 2.6 and 3.9 months [25, 33] with a total range from 1.5 to 7.0 months [22, 35]. Average patient weight was reported in only 2 (18.2%) articles, given by 6.3 ± 2.1 kg [24] and 5.4 kg (range: 3.8–6.1 [35]).

Table 1 provides an overview of the patient sample.

**Table 1:** The patient sample of each analyzed publication within its age and sex

Reference	Patients (*n*)	Age at surgery (months)	Sex (*n*=)
Bonfield, 2018 [6]	5	n.g.	n.g.
Brown, 2011 [8]	52	$\overline{X}$ 3.08	n.g.
Iyer, 2017 [24]	7	$\overline{X}$ 15.2 ± 7.7* $\overset{\sim }{x}$ 12.3*	6 ♂ 1 ♀
Iyer, 2018 [25]	31	$\overline{X}$ 2.7 *r* (1.6–3.2)	27 ♂ 4 ♀
Isaac, 2018 [22]	187 (207 in total; 187 endoscopic vs. 20 undergone CVR; data separated in article)	$\overset{\sim }{x}$ 3.0 IQR [2.5–4.0] *r* (1.5–7.0)	137 ♂ 50 ♀
Jimenez, 2012 [28]	256	$\overline{X}$ 3.9	187 ♂ 69 ♀
Lepard, 2021 [33]	19 (50 patients in total; 19 endoscopic vs. 31 undergone open surgical correction; data separated in article)	$\overset{\sim }{x}$ 2.6 *r* (2.4–2.9)	16 ♂ 3 ♀
Magge, 2019 [34]	30 (51 in total, 30 endoscopic vs. 21 undergone pi-procedure; data separated in article)	$\overline{X}$ 3.11 (± 3.18)	n.g.
Martin, 2018 [35]	5	$\overline{X}$ 2.8 *r* (1.5–4.5)	3 ♂ 2 ♀
Nguyen, 2017 [37]	100	$\overline{X}$ 3.3 (± 1.1)	70 ♂ 30 ♀
Ridgway, 2011 [39]	56	$\overline{X}$ 3.24 (± 1.48)	47 ♂ 9 ♀
Schulz, 2021 [42]	17 (128 in total: sagittal CS (*n* = 17) with endoscopic treatment vs. conventional/open surgery (*n* = 29); metopic CS with endoscopic treatment (*n* = 16) vs. conventional/open surgery (*n* = 18); non-affected control groups at 6 (*n* = 30) and 24 (*n* = 18) months of age)	$\overset{\sim }{x}$ 3.0 *r* (2.1–3.9)	12 ♂ 5 ♀
Shah, 2011 [44]	47 (89 in total; 47 endoscopic vs. 42 CVR; data separated in article)	$\overline{X}$ 3.6	32 ♂ 15 ♀
Wood, 2017 [48]	73	Group A: *X̅* 2 Group B: *X̅* 2.7	n.g.

Characteristics: $\overline{X}$ mean, $\overset{\sim }{x}$ median, *IQR* interquartile range, *r* range, *n.g.* not given

*Weeks

In the selected publications, reporting cases or case series of children with single suture craniosynostosis treated via endoscopic or endoscopically assisted approaches, we identified 5 key steps in the management of patients and aimed to present the different methods reported by our colleagues in their publications within each step and provide data on how often each variant is used within our sample.

## General considerations

There is general agreement in putting much emphasis on a multidisciplinary workup. We looked for suggested team constellations described in the data, as well as related tasks each one of the team members had. Nine series stated their procedures were carried out by a single neurosurgeon [8, 22, 24, 25, 28, 32, 33, 38, 47]. In 2 series, the decision for one or more additional craniofacial/plastic surgeons or an accompanying pediatric neurosurgery fellow to participate depended on the severity of the underlying pathology [6, 35, 37, 44]. An anesthesiologist was mentioned to work solely for the group, responsible as a pediatric specialized member, in 2 reports [8, 35].

### Step 1: Clinical examination and preoperative diagnostics

The majority of the groups indicated carrying out a clinical examination [6, 8, 22, 25, 28, 39, 42, 48]. If not performed routinely, the reasons for ophthalmologic investigation mainly were not only to further evaluate the intracranial pressure by screening for papilledema but also to keep track of possible deviations in line of sight [22]. Our analyzed sample did not provide any detailed information on the neuropsychological investigation [31] applied. However, several of the remaining articles emphasized that dedicated maintenance of patients including their caregivers would be crucial to survey both the neurocognitive status and the development of affected children, as well as the stress and burden the patients and their families had to deal with [37, 44].

A cooperating geneticist was usually consulted on-demand either in case of clinical signs that likely indicate a syndromic etiology or in existence of a conspicuous own, respectively, family anamnesis [39, 44]. Having reviewed the published insights gained over the past 15 years within the use of molecular tools (e.g., whole-genome sequencing), Armand et al. [3] concluded that the availability of such blurred the limit between syndromic and non-syndromic craniosynostosis (CS) even further emphasizing the genetic heterogeneity of these conditions. Shedding some light on darkness, the group proposed a diagnostic flowchart with indications for a systematic molecular assessment in patients with CS.

Reporting on their clinical examination, five authors supplemented such with a CT scan [22, 28, 34, 37, 44]. Among them, four indicated to do so in all patients [28, 34, 37, 44], whereby only one [22] preserved the confirming method for cases of observed papilledema or macrocephaly [22]. Other teams elaborated their anthropometric measurements by caliper [25, 39, 44], either using existing 3D reconstructed CT scans [34, 37, 44], laser scans evaluated by the orthotist [2, 11, 25, 33, 35, 42, 48], or their results on 3D images taken photographically [39]. The most common anthropometric measurement tool used in our included data was the CI (cranial index, defined by BP/AP diameter × 100). Isaac et al. [22] applied it as the “*Z*-score” (e.g., obtained CI sample values were standardized using age- and sex-matched normative data), and Schulz et al. [33] examined an own non-affected control group for concrete morphometric comparison, whereas the remaining authors chose the more standard version mentioned above.

Table 2 summarizes the examination tools used by our sample.

**Table 2:** An overview of the type of examinations and measurements each group analyzed performed

Examination/measurement	Reference
CT scan	Isaac, 2018 [22] Jimenez, 2012 [28] Nguyen, 2017 [37] Magge, 2019 [34] Shah, 2011 [44]
3D/laser scan	Brown, 2011 [8] Iyer, 2018 [25] Jimenez, 2012 [28] Lepard, 2021 [33] Martin, 2018 [35] Nguyen, 2017 [37] Schulz, 2021 [42] Wood, 2017 [48]
“Head measurements”	Brown, 2011 [8] Lepard, 2021 [33] Magge, 2019 [34] Martin, 2018 [35] Schulz, 2021 [42]
CI	Iyer, 2017 [24] Iyer, 2018 [25] Isaac, 2018 [3] Jimenez, 2012 [28] Lepard, 2021 [33] Magge, 2019 [34] Martin, 2018 [35] Nguyen, 2017 [37] Ridgway, 2011 [39] Schulz, 2021 [42] Shah, 2011 [44] Wood, 2017 [48]
HCP	Isaac, 2018 [22] Lepard, 2021 [33] Ridgway, 2011 [39]
Ultrasound examination	Bonfield, 2018 [11]
Clinical examination	Bonfield, 2018 [11] Brown, 2011 [8] Iyer, 2018 [25] Isaac, 2018 [22] Jimenez, 2012 [28] Ridgway, 2011 [39] Schulz, 2021 [42] Wood, 2017 [48]
Fundoscopy/ophthalmological examination	Isaac, 2018 [22] Shah, 2011 [44]
Neurocognitive evaluation	Nguyen, 2017 [37] Shah, 2011 [44]
Genetic investigation	Ridgway, 2011 [39] Shah, 2011 [44]
Subjective rating for “normalcy of craniofacial appearance” after standardized photography (Likert Scale 1–5, rated by multiple independent groups)	Lepard, 2021 [33]
Intracranial monitoring (ICP)	Isaac, 2018 [22]

#### Indication for surgery

Indication for surgery was placed by head measurements, radiographs, and/or CT scans following the clinical examination [2]. Apart from the medical advocacy, the ultimate informed decision on surgery type was taken by the caregivers in each case. Corresponding to the common prevalent standard of care [27, 32, 40], all groups analyzed recommended endoscopically assisted craniosynostosis surgery (EACS) with postoperative helmet therapy (PHT) at least for non-syndromic sagittal CS children < 3 months of age, equally. However, several authors expanded the formerly mentioned time frame to 4–5 months [2, 39], occasionally to 6–7 months [35], or even up to 9 months [22] for selected cases of milder deformities. After having operated on children even > 12 months of age, Shah et al. [44] resumed that an endoscopic technique in isolated sagittal CS would be difficult to perform in patients having passed 6 months of age due to bone thickness and moreover be less efficacious in children older than 3 months.

### Step 2: Perioperative workup

Lines inserted comprised one or two intravenous lines [2, 25, 28, 35, 37] and an additional arterial line, if possible [25]. Two authors [2, 37] referred to have used arterial and/or central venous catheter lines at the beginning when EACS has been implemented at their institution but would have eliminated those shortly after recognizing they could achieve good results and did not obligately need extra lines to keep their patients safe. The placement of temperature probes was alluded to in one article [2]. Besides routinely applied intraoperative monitoring tools, such as an electrocardiogram, a noninvasive blood pressure cuff, and pulse oximetry, the use of precordial duplex ultrasound was introduced [2]. Concerning preoperative blood work, only one article presented their routine as a single heel stick hematocrit once the patient was under general anesthesia [28]. One group employed the use of a pre-procedure bolus of 10 mg/kg tranexamic acid (TXA) followed by an infusion of 5 mg/kg/h in all of their patients [35]. Evidentially, perioperative administration of antibiotics is crucial. This point was either just listed as “intravenous antibiotics” [2, 28] or specified as intravenously administered cefazolin within 30 min [2] or 60 min [28] prior to skin incision. One of the surgeons prescribed two additional IV doses of antibiotics to be administered every 8 h after premedication [2].

### Step 3: Surgery

Following induction with inhalation anesthetics [2], patients typically received endotracheal intubation [2, 28, 35, 37]. In one publication, dexamethasone was reported to be given before skin incision to allow for early helmet application on postoperative day (POD) 1 [37].

Despite one group investigating supine positioning in scaphocephalic patients to minimize the risk of venous air embolism [35], positioning was uniformly conducted prone by all remaining groups, which labeled it either as modified/ prone [2, 22, 39, 48], sphinx [6, 28, 37, 44], or seal [24] position. Prone positioning was realized either on chest rolls fixing the head in a Pro Med DORO multipurpose skull clamp [24, 37, 39] combined with U-shaped gel supports to cup the ears [2, 34] or a bean bag with the neck in extension.

Right before skin incision, the scalp was infiltrated with a combined 0.25% bupivacaine solution and 1:200,000 epinephrine locally at the incision site [2, 39]. Possible difficulties to find any landmarks, such as the lambda, could be addressed via ultrasound, verifying the correct position and marking it [6]. Either a Colorado needle [39] or a #15 knife blade was chosen to incise the skin, whereafter a needle tip on the electrosurgical unit was used for subgaleal dissection [2]. The majority of surgeons performed two transverse skin incisions. In contrast, Iyer et al. [24] developed a technique with a single, transverse 3-cm incision about 1 cm posterior to the anterior fontanelle — later also realized by Schulz et al. [42], following which they left the pericranium intact to mark the bone using electrocautery. Burr holes were performed using a high-speed drill [2, 24, 35, 39, 48] and hereafter locally enlarged with Kerrison rongeurs [2, 22, 35, 39]. After providing access for the endoscope into the epidural space, the suturectomy was accomplished using bone-cutting scissors [2, 28, 35, 37, 44, 48], bone-cutting rongeurs, and high-speed drills. The width of the suturectomy varied between the groups.

Similar strategies have been pursued to achieve hemostasis. Above all, of course, electrocautery and bone wax were commonly applied. [2, 24, 28, 44, 48]. Since suction cautery devices (i.e., Bovie suction, Valley lab; [28, 44]) and piezoelectric tools [24] have found increasing acceptance in the field over the last few years [9], several groups in our sample appreciated the advantages of such [24, 28, 44]. Thrombin was introduced in varying forms — either as thrombin-soaked gel foam [2, 24, 39], in its injectable consistency [35] fabricated as Floseal [37], or Surgiflow [28]. Before wound closure, two teams used antibiotic irrigation (Wood [48], Ridgway [39]), e.g., with bacitracin [39]. Galeal closure was commonly performed with absorbable sutures [2, 24, 39], which was also the material of choice for dermal closure [2, 24, 28, 35, 37, 39, 48]. Others used skin glue either solely or in addition to their absorbable suture [24, 28]. Average operating time varied widely between the groups depending on the severity of deformation and the surgeon’s experience.

Table 3 provides an overview of the perioperative parameters investigated.

**Table 3:** The perioperative parameters investigated in our patient sample

Reference	Surgical incisions	Craniectomy	Average operating time (min.)	Blood loss (ml)	Transfusion rates (%)	Perioperative complications
Bonfield, 2018 [6]	2 incisions, 2–4 cm in length^A^; verification of lambda via ultrasound prior to prepping and draping	n.g.	n.g.	n.g.	n.g.	n.g.
Brown, 2011 [8]	2 incisions, 2 cm in length, placed perpendicular to the midline^A^	2–3 cm in width^B^	$\overline{X}$ 44.7	n.g.	7	n.g.
Iyer, 2017 [24]	1 single, transverse incision 1 cm posterior to the anterior fontanelle, 3 cm in length	3 cm in width^B^	$\overline{X}$ 87 ± 10.5	$\overline{X}$ 32 ± 13.5	0	1 patient with 2 small dural tears repaired primarily
Iyer, 2018 [25]	n.g.	3 cm in width^B^; in early experience barrel staves were added, later not	n.g.	n.g.	n.g.	n.g.
Isaac, 2018 [22]	2 incisions, 2 cm in length, placed perpendicular to the fused sagittal suture^A^	1 cm in width^B^	$\overset{\sim }{x}$ 45 *r*: 39–52	$\overset{\sim }{x}$ 25 *r*: 20–30	2	- 2 surgical site infections (1%) that necessitated debridement - 1 superficial wound infection in treated with oral antibiotics
Jimenez, 2012 [28]	2 “small incisions” crossing the midline^A^	Wide-vertex craniectomy with bilateral barrel stave osteotomies; width was inversely proportional to the baby’s age; very young infants: 5–6 cm, older children: 2–3 cm	$\overline{X}$ 57	$\overline{X}$ 27	7	n.g.
Lepard, 2021 [33]	n.g.	n.g.	$\overset{\sim }{x}$ 80.5 *r*: 75.5–86.3	$\overset{\sim }{x}$ 20 (8.7–31.3)	10.5	n.g.
Magge, 2019 [34]	2 small incisions^A^	1-2 cm strip craniectomy^B^	$\overset{\sim }{x}$ 69.5 ± 10.3	$\overline{X}$ 35.0 ± 29.0	16.7	None
Martin, 2018 [35]	2 transverse incisions^A^	“narrow vertex suturectomy”	$\overline{X}$ 70 *r*: 65–81	n.g.	0	n.g.
Nguyen, 2017 [37]	2 transverse midline incisions^A^	4–5 cm in width^B^; performed in conjunction with bilateral wedge osteotomies	$\overline{X}$ 77.1 ± 22.2	$\overline{X}$ 34.0 ± 34.8	9	- 1 conversion to open technique required due to presence of a large emissary vein difficult to control endoscopically; - 1 readmission due to emesis while a small SAH occurred - Postoperative period: hyperglycemia, respiratory distress requiring CPAP, respiratory syncytial virus infection
Ridgway, 2011 [39]	2 incisions, 1.5–2.5 cm in length^A^	1 cm strip craniectomy^B^	$\overline{X}$ 45.32 ± 11.23	n.g.	3.6	None
Schulz, 2021 [42]	“1 small skin incision”	2–3 cm strip craniectomy^B^	n.g.	n.g.	n.g.	n.g.
Shah, 2011 [44]	2 incisions^A^	4–5 cm^B^; bilateral osteotomies were added	$\overline{X}$ 88	*X̅* 29	6.4	None
Wood, 2017 [48]	2 incisions, 2–3 cm in length^A^	Center A: 1–2 cm^B^ Center B: 3 cm^B^; barrel staves were added	Cente*r* A: $\overline{X}$ 71.6 Cente*r* B: $\overline{X}$ 111	Cente*r* A: $\overline{X}$ 38 Cente*r* B: $\overline{X}$ 44.5	n.g.	n.g.

$\overline{X}$ mean, $\overset{\sim }{x}$ median, *r* range, *n.g.* not given

^A^Placed in the standard localization posterior to the anterior fontanel and anterior to lambda

^B^In total length of the sagittal suture

### Step 4: Postoperative management

#### Wound dressing

The postoperative dressing was further described by Wood et al. [48], who placed an abdominal pad over the top of the patient’s head along the suture removal site and secured it with stretchable netting adding some pressure to decrease postoperative venous bleeding for approximately 8 h.

#### Postoperative monitoring and pain management

Lying in the postoperative crib, the head of the bed elevated about 30 to 45°, the patients were transported either to the postoperative care unit (PACU) [2], to the pediatric intensive care unit (ICU) [37, 44], or directly to a standard surgical ward [28]. Nguyen et al. [37] changed their protocol for extubated patients after the first 3 years from former transfer to the ICU to the latter management directly on the surgical ward without invasive monitoring. For analgesia, the typical protocols scheduled acetaminophen to be applied rectally supplemented with IV morphine as needed [2] or alternated acetaminophen orally with ibuprofen every 3 h with IV morphine every hour as needed for breakthrough pain [28]. At that point, Jimenez et al. [28] introduced their observation on their patients typically settling down and requiring minimal pain medication after having passed the first 8 h following surgery. Diet then was advanced as tolerated, while breast-fed infants were allowed to be nursed immediately after surgery [28].

#### Management of blood loss and transfusion rates

Regarding blood loss, the analyzed sample did not vary a lot, referring to median estimated blood loss values from 20 ml [33] up to 44.5 ml [50, group B] regardless of the width of suturectomy. Though detailed protocols for cHb or Hct measurements were rare in the analyzed sample, we could identify one group that obtained the first heel stick hematocrit right before surgery followed by a second test on postoperative day (POD) 1 prior to discharge [28]. Another group checked the hematocrit at the conclusion of surgery to assess and estimate the blood loss [2]. Regimens for blood transfusion were described insofar as only symptomatic patients (i.e., tachycardic, hypotensive) were being transfused, whereby there occasionally was a mentioned threshold alongside hematocrit levels, for example, lower than 20% [2, 24]. Introducing their newer transfusion protocol, which required a hematocrit level of 18.0% or less, one group mentioned looking into the method of preoperative erythropoietin (EPO) use in addition to their current intraoperative standard [44]. At some centers, the parents or rather family members were given the opportunity of a preoperative donor-directed blood donation for their affected young relative [2, 44].

#### Length of hospital stay

Length of hospital stay (LHS) has been shown to be very similar in the series indicating patients’ discharge after one day on average. The reported complications, occasionally delaying the mentioned period, consisted of dural tears [24], surgical site infections [22], as well as conversion to an open technique [37]. Moreover, readmission due to a small postoperative subarachnoid hemorrhage (SAH) [37], as well as non-compliance with helmet therapy in one group [22], was mentioned.

### Step 5: Follow-up

Concerning follow-up, six reporting surgeons saw their patients during this longitudinally process themselves [2, 22, 25, 34, 39, 48]. An orthotist was involved in every group. Among these, he or she was either exclusively responsible for the postoperative molding therapy [39, 48] or also for the preoperative assessment of arithmetic measurements to support in grading an underlying pathology and collecting comparative data over time [2, 25, 35, 37, 44]. The average time of follow-up varied widely among the analyzed groups as we could identify a total span from a median of 13 months [44] over a median time of 2.8 years (range: 1.0–5.2 years) [25], extending to a reported maximum of 15 years [28] in our sample. An accompanying orthotist performed follow-up imaging in 3 cases [2, 39, 44], whereby visiting took place approximately every 2–4 weeks until the attending physician discontinued helmet therapy. The surgeon was commonly consulted in a frequency of about 2–3 months [2, 39, 44]. This variety of data underlines that a consistent analysis and comparison between the different groups is impossible as long as there is no guideline protocol for evaluation in these children.

#### Helmet therapy

Postoperative helmet therapy (PHT) was mainly initiated within postoperative week one [2, 22, 24, 28, 33–35, 39, 42, 44] after individual evaluation, e.g., by an orthotist using an infrared beam STARscanner [28, 33]. In some teams, this assessment took place even preoperatively [35, 37] to start with orthotic therapy as soon as possible. Once fitted, the helmet was worn for ~ 23 h/day in all cases referring to that point, and only put down for washing, care, and by doing so checking the skin for bruises, exfoliative or erosive issues, etc. The average length of molding therapy varied from 5.0 (range: 2.9–14.5) months [42] up to 1 year [28]. Listed criteria for planned discontinuation of orthotic therapy comprised either achieving a “normalized” cranial shape [2] or the child completing its first ten months [2] respective the first year [22, 25, 37, 44] of age. Furthermore, therapy was stopped prior to 1 year of age if either the desired phenotype and some overcorrection of the CI were obtained [39] or treatment for a minimum of 6 months was accomplished with achieved goals of a CI > 0.8 and/or parental satisfaction with the aesthetic result [33]. Interesting findings were published by Iyer et al. [25], who presented a rare comprehensive analysis of an optimal duration of PHT and its influencing factors. By evaluation of the CI in a rigid pattern (preoperatively, at its peak level, termination of helmet therapy, and last follow-up), they could show that patients undergoing endoscopically assisted craniosynostosis surgery (EACS) and PHT for sagittal synostosis reach a peak CI around 7 to 9 months after surgery which does not improve beyond CI_max_ despite the continuation of PHT. Moreover, all patients showed a retraction of CI at the last follow-up, from an average of 0.83 ± 0.01 at CImax to 0.78 ± 0.01 at CIfinal.

#### Evaluation of outcome

Assessment tools for outcome measurements included 3D laser/infrared scans [2, 25, 33, 35, 42, 48] obtained during the frequent visits to the collaborating orthotist. In contrast, one author explained using available pre- and postoperative low-dose radiation CT scans to determine changes in CI [37]. Evaluating outcomes additionally on a subjective basis, Lepard et al. [33] performed standardized full-face photography of their patients including top, anterior, and lateral views, which were subsequently rated by independent faculty surgeons, surgical trainees, nurses, and laypersons for the normalcy of craniofacial appearances using a 5-point Likert scale.

All of our sample’s authors assessed the CI or “cranial measurements” as outcome parameters within their follow-ups [22, 24, 25, 28, 35, 37, 44, 48]. The cranial index was usually presented as a mean (±SD) or median value, while one group provided their CI data in categories within classifying a CI of >80 as excellent, 80–70 as good, and <70 as poor [28]. Isaac et al. [22], applying their *Z*-score for CI measurement at years 1, 2, and 3 each following surgery, also added the head circumference percentile (HCP) and rated aesthetic outcomes using the Whitaker classification. Herein, most patients were associated with Whitaker class I (99%), i.e., no surgical reoperations were considered necessary by the surgeon, the patient, or the family. Thus, the minor part (1%) of the patients was categorized in class III, i.e., major secondary osteotomies or grafts had to be performed. However, the Whitaker classification remains a subjective grading tool lacking comparability in the pursuit of independent standardization. A monocentric, longitudinal study by Wes et al. [47] has shown that the Whitaker classification exhibits low inter-rater reliability and does not predict future treatment. Nonetheless, they considered the scheme to be helpful in creating new evaluation tools with greater precision to improve the quality of patient care and outcome research.

Due to the persistent lack of tangible, consistent assessment tools relating adequately to this patient group, Szpalski et al. [46] proposed a list of longitudinal parameters of care that might be considered in terms of evaluation, treatment, and following-up on patients with craniosynostosis. In order to capture the possible associated impairments — particularly in syndromic or complex cases — in a reasonable way, those suggested items contained the “Bayley Scales of Infant Development,” the exploration of oral, ophthalmic, and ear-nose-throat conditions, as well as the parental satisfaction with the patient’s appearance and “HR/QOL” (health related/quality of life) to give just a brief insight.

## Discussion

### General considerations

A team approach allows to manage the different aspects more effectively and comprehensively and should be aimed for whenever possible. A craniosynostosis specialized team could comprise a pediatric neurosurgeon, an accompanying oral and maxillofacial surgeon, a pediatric anesthesiologist, a pediatrician, and a trusted orthotist. Pediatric neuropsychologists, ophthalmologists, dentists, otolaryngologists, and geneticists should be consulted as required.

In line with our colleagues [11, 27, 41], we consider patients 3 months of age as ideal patients for EACS followed by helmet therapy. Although the results seem to be less ideal for older children, decisions are formed individually and caregivers should be informed about the possible surgical options and expected outcome and take the decision. In less severe cases or in cases in which a child is simply referred too late, children up to 7 months can be considered for minimally invasive approaches.

### Preoperative examination

The sagittal suture can be adequately explored for closure or patency within the use of ultrasound in almost all cases. In a strive to minimize the patients’ exposure to radiation whenever possible [43], the performance of routine CT scans pre- or postoperatively has been abandoned by most colleagues. 3D dimensional photographs, in addition to the typical scores (CI, etc.) mentioned before, are valuable to guide the postoperative therapy and evaluate and compare the outcome more objectively [31]. Underpinning this ratio, the team among Schweitzer [43] regarded CT scans and plain radiographs not to add essential information upon an extensive clinical examination in most of the cases in single suture craniosynostosis. Consequently, they resorted to ultrasound examination to determine the state of the sutures and drew back on CT scans just in case of remaining diagnostic doubts, which only occurred in 2 of their 137 patients presented.

A CS-standard next-generation sequencing (NGS) panel for routine genetic analysis [3, 23, 49] might be used in single suture synostosis without expecting any relevant abnormalities. Only in cases of suspected syndromological status an extended gene panel is strongly recommended.

To rule out an elevated intracranial pressure, a standardized neurological examination as well as a general ophthalmological examination are regarded sufficient by most colleagues. Additional diagnostic steps relating to suspected elevated intracranial pressure and concomitant ophthalmologic deviations, such as V-pattern strabismus, ocular torticollis, aniso-astigmatism, or any dental-maxillary or auditive disturbances as well as further genetic disorders, subsequently are initiated whenever assumed necessary in consultation with the particular specialists.

**Fig. 2:**
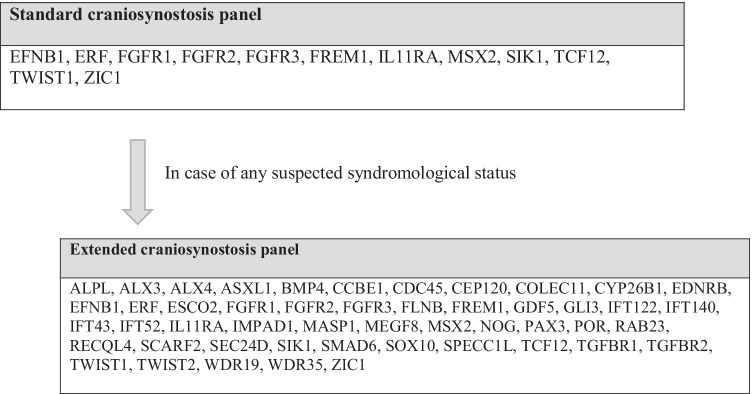
Genetic investigation scheme used for craniosynostosis patients at our institution

Figure 2 shows the genetic investigation scheme used for craniosynostosis patients at our institution.

### Perioperative workup

To address the problem of the physiologic nadir that typically comes along with children treated for craniosynostosis at the appropriate age, resulting in low levels of hemoglobin ~ 8 mg/dl as a starting point before surgery, blood loss has to be reduced to a minimum. Therein, thromboxane (TXA) has been shown to be beneficial to significantly decrease the number and volume of packed red blood cell transfusions and the rate of transfusions needed in children undergoing CS surgery while providing excellent patient tolerance [7, 14, 45]. Although patients might need an intraoperative blood transfusion, two peripheral venous lines and one arterial line, a temperature probe, and a urinary catheter for output control are deemed sufficient by most authors. A precordial duplex alongside routine intraoperative monitoring (ECG, BP, respiratory function monitoring, pulse oximetry) can be used to detect air embolism. Blood gas analysis, including cHb, should be checked before, during, and after surgery.

### Surgery

As there are no studies comparing different variations of surgical techniques, it remains difficult to argue in favor of one over another and ultimately remains in the hands of the surgeon and his or her preferences and experience to determine the procedure.

Although it is not part of the series, we reviewed it is important to mention that a relevant number of colleagues perform spring-assisted surgery in which stainless-steel springs are placed in the osteotomy gap. This technique is based on the principle of distraction osteogenesis and uses the distracting force of the springs (mean 6.9 N) to guide cranial growth. It is important to emphasize that a second procedure is required to remove the springs. Despite some specific implant related complications, the results reported by various groups are similar to what can be achieved with open or endoscopic procedures and PHT [30, 40].

In our analysis, all but one group used the prone position which offers comfortable access to the sagittal suture. To lower the risk of air embolism and pressure related injuries, we prefer to position our patients supine with the head turned right as described by Martin et al. [35].

Garland et al further analyzed surgical techniques for sagittal craniosynostosis [16] and found significant variations for incisions and osteotomies. While some surgeons believe a small single strip suturectomy is sufficient, others add lateral paramedian osteotomies posterior to the coronal suture and anterior to the lambdoid sutures to aid the remodelling. According to former groups including the one among Magge et al. [33] and Wood et al. [48] who could previously demonstrate that the addition of barrel staves would not be necessary for the correction of sagittal craniosynostosis, we do not routinely implement this surgical step either but think it might be helpful in selected cases.

The width of the osteotomy also varies significantly, while Ridgeway et al. propose a narrow suturectomy of 1 cm others extend the osteotomy up to 5 cm and add the described wedge osteotomies.

As described by Bonfield et al. [6], ultrasound might help to identify and mark the posterior and anterior fontanelle to avoid any possible uncertainties.

As blood loss is one of the main risks of the procedure, infiltrating the skin with combined 0.25% bupivacaine solution and 1:200.000 epinephrine locally before skin incision is recommended by most surgeons. A supraperiostal preparation might help to reduce bleeding as well. To achieve good hemostasis and to proceed with the surgery in a safe manner, we appreciate the introduction of an ultrasonic aspiration device (SONOPET®), coagulation suction, and silicon-coated spatulas. The very common use of bone wax should be kept only to the extend particularly needed to avoid the risk of producing bone tissue necrosis.

Tisseel should be inserted with caution as there are case reports [15] on associated venous air embolism suspecting that application at a close distance into an endoscopic canal may have resulted in air being forced into the venous sinuses. What might be helpful to circumvent this possible risk is to apply the product slowly from a larger distance while paying increased attention to the child’s monitor including precordial duplex to detect this complication immediately.

In addition to that, concerning the minimization of transfusions, many performing surgeons [8, 28, 32, 48] reported on a learning curve over time which leads to shorter operation times, reduced blood loss, and less number of transfusions needed as a result during the endoscopic procedure, on which we agree upon in our experience.

### Postoperative management

While some authors report that their patients are handed to the pediatric ICU, where they usually stay for one day, other groups report that their patients are handled on a regular ward. The decision if an ICU is required might largely depend on the structure and organization of the hospital and does not seem to have an impact on complication rates and outcome.

The decision on blood transfusion is mostly set on a case-by-case basis, whereby we predefined a threshold of < 8 mg/dl Hbc in combination with hemodynamic instability as reference points in our protocol. In most cases, patients get a postoperative 3D scan (for PHT planning and outcome observation) right before being discharged. As mentioned before, a routinely performed postoperative CT scan should be avoided to reduce radiation.

Dural tearing is one of the most common surgical complications in terms of non-syndromal single-suture synostosis according to the current literature [4]. Such, as well as further perioperative complications, probably might be underrepresented in our sample considering the apparently low complication rate shown. Presumably, this might be attributable to differing scopes of the studies examined, which were defined instead by, e.g., evaluation of PHT or outcome measurement tools. Nevertheless, if there might be drawn a conclusion when it comes to secure performance of EACS, we think that only by close communication between surgeons and anesthesiologists using predefined workflows, thresholds (e.g. for blood transfusion), and clear evasive strategies in case of unexpected perioperative complications, predictable and safe handling of the youngest can be ensured. Furthermore, we agree with Iyer et al. [24] who explained the occurrence of dural tears could be largely eliminated since cottonoids were placed underneath the piezo device.

### Follow-up

As cases, techniques, and logistics differ significantly, it seems difficult or rather pointless to make very detailed recommendation concerning the follow-up. The follow-up appointments at our center are usually set in 4–8-week intervals, while helmet therapy is commonly continued for 10–12 months postoperatively. If the helmet therapy is discontinued too early, there is a risk of a certain relapse while continuing the therapy for too long seems not to further improve results. HCP, CI, oblique cranial length (OCL = frontozygomaticus-contralateral euryon, for each side), and forehead inclination should be routinely observed as longitudinal outcome parameters, while the primary goal is to achieve parental and professionals’ satisfaction. The patients’ neurocognitive matters as well as their caregivers’ quality of life (QoL) should be assessed whenever possible as these are relevant outcome parameters for comparing different surgical techniques. The Bayley Infant Neurodevelopmental Screener (BINS) and the Infant Toddler Quality of Life (ITQoL) are routinely used.

In our opinion, knowing the patient’s holistic social and medical constellation as well as bringing a deep understanding of the consequences of a molding procedure are paramount aspects to provide a successful helmet therapy. The PHT is an essential part of the effort to normalize the patients’ head shape. We, therefore, believe it should be led by the treating physicians rather than being completely outsourced to a third party.

**Fig. 3:**
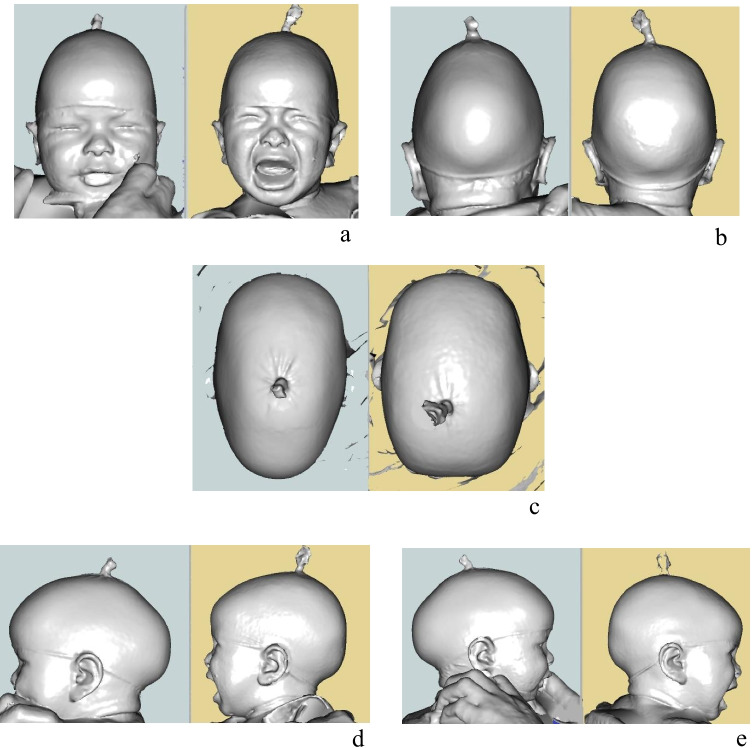
3D images taken of one of our patients pre- and at 5 months postoperatively. **a** The frontal, **b** the rear, **c** the top, **d** the left lateral, and **e** the right lateral views. The preoperative status is demonstrated on the left in each view (in blue) and the 5-month postoperative view on the right (in yellow)

Figure 3 shows 3D images taken of one of our patients pre- and postoperatively over time.

## Conclusion

There exist significant variations in surgical techniques and patient management for children treated endoscopically for single suture sagittal synostosis, which is reflected in the published series. In particular, this leads to a diverging presentation of the according data analyzing multiple different aspects. As often stated over the past years [1, 11, 19, 27, 45], this heterogeneity constitutes a major problem in terms of comparability between different strategies.

## Data Availability

Not applicable.
